# Allyl Isothiocyanate (AITC) Induces Apoptotic Cell Death In Vitro and Exhibits Anti-Tumor Activity in a Human Glioblastoma GBM8401/*luc2* Model

**DOI:** 10.3390/ijms231810411

**Published:** 2022-09-08

**Authors:** Kung-Wen Lu, Tai-Jung Lu, Fu-Shin Chueh, Kuang-Chi Lai, Te-Chun Hsia, Shu-Fen Peng, Ching-Chang Cheng, Yu-Cheng Chou, Fei-Ting Hsu

**Affiliations:** 1 College of Chinese Medicine, School of Post-Baccalaureate Chinese Medicine, China Medical University, Taichung 404, Taiwan; 2 Department of Biological Science and Technology, China Medical University, Taichung 404, Taiwan; 3 Department of Food Nutrition and Health Biotechnology, Asia University, Taichung 413, Taiwan; 4 Department of Medical Laboratory Science and Biotechnology, College of Medical Technology, Chung Hwa University of Medical Technology, Tainan 717, Taiwan; 5 Department of Surgery, China Medical University Beigang Hospital, Beigang, Yunlin 651, Taiwan; 6 Department of Respiratory Therapy, China Medical University, Taichung 404, Taiwan; 7 Department of Internal Medicine, China Medical University Hospital, Taichung 404, Taiwan; 8 Department of Medical Research, China Medical University Hospital, Taichung 404, Taiwan; 9 Director of Laboratory Animal Service Center, Office of Research and Development, China Medical University, Taichung 404, Taiwan; 10 Department of Neurosurgery, Neurological Institute, Taichung Veterans General Hospital, Taichung 407, Taiwan; 11 Department of Applied Chemistry, National Chi Nan University, Nantou 545, Taiwan; 12 Department of Post-Baccalaureate Medicine, College of Medicine, National Chung Hsing University, Taichung 402, Taiwan

**Keywords:** AITC, glioblastoma GBM8401/*luc2* cancer cells, xenograft, caspase, apoptosis

## Abstract

Some clinically used anti-cancer drugs are obtained from natural products. Allyl isothiocyanate (AITC), a plant-derived compound abundant in cruciferous vegetables, has been shown to possess an anti-cancer ability in human cancer cell lines in vitro, including human brain glioma cells. However, the anti-cancer effects of AITC in human glioblastoma (GBM) cells in vivo have not yet been examined. In the present study, we used GBM8401/*luc2* human glioblastoma cells and a GBM8401/*luc2*-cell-bearing animal model to identify the treatment efficacy of AITC. Here, we confirm that AITC reduced total cell viability and induced cell apoptosis in GBM8401/*luc2* cells in vitro. Furthermore, Western blotting also showed that AITC induced apoptotic cell death through decreased the anti-apoptotic protein BCL-2, MCL-1 expression, increased the pro-apoptotic protein BAX expression, and promoted the activities of caspase-3, -8, and -9. Therefore, we further investigated the anti-tumor effects of AITC on human GBM8401/*luc2* cell xenograft mice. The human glioblastoma GBM8401/*luc2* cancer cells were subcutaneously injected into the right flank of BALB/c nude mice to generate glioblastoma xenograft mice. The animals were randomly divided into three groups: group I was treated without AITC (control); group II with 0.1 mg/day of AITC; and group III with 0.2 mg/day of AITC every 3 days for 27 days. Bodyweight, and tumor volume (size) were recorded every 3 days. Tumors exhibiting Luc2 intensity were measured, and we quantified intensity using Living Image software on days 0, 12, and 24. After treatment, tumor weight from each mouse was recorded. Tumor tissues were examined for histopathological changes using H&E staining, and we analyzed the protein levels via immunohistochemical analysis. Our results indicate that AITC significantly inhibited tumor growth at both doses of AITC due to the reduction in tumor size and weight. H&E histopathology analysis of heart, liver, spleen, and kidney samples revealed that AITC did not significantly induce toxicity. Body weight did not show significant changes in any experiment group. AITC significantly downregulated the protein expression levels of MCL-1, XIAP, MMP-9, and VEGF; however, it increased apoptosis-associated proteins, such as cleaved caspase-3, -8, and -9, in the tumor tissues compared with the control group. Based on these observations, AITC exhibits potent anti-cancer activity in the human glioblastoma cell xenograft model via inhibiting tumor cell proliferation and the induction of cell apoptosis. AITC may be a potential anti-GBM cancer drug that could be used in the future.

## 1. Introduction

Highly angiogenic brain tumors are the most frequent and lethal cancers of the human central nervous system. Glioblastoma multiforme (GBM), a grade-IV astrocytoma and one of the deadliest cancers [1], is the most biologically aggressive subtype, and is a tumor with a dismal prognosis [2]. It has a median survival period of 14.6 months from diagnosis [2,3]. Currently, the standard treatment of GBM patients is surgical resection. Following this, patients are treated with adjuvant radiation therapy and chemotherapy with oral DNA-alkylating temozolomide (TMZ), which elevates the median survival period only to 14.7–15 months [4,5,6]. Moreover, GBM manifests resistance to standard therapy, such as radiotherapy-induced alterations, leading to GBM recurrence and aggressiveness dispersed into the surrounding normal brain [7]. After treatment, the survivors showed significant long-term debilitating side effects. Thus, finding new compounds from natural products is one strategy for reducing side effects in GBM patients.

Several reports have indicated that the plant-derived compounds possess chemopreventive and anti-cancer activities [8,9,10]. Isothiocyanates (ITCs) are found in broccoli, cabbage, cauliflower, and Brussels sprouts that belong to Cruciferae, a family of vegetables that present protection against cancer development [11,12]. Allyl isothiocyanate (AITC), one of the constituents of naturally occurring ITCs, and thus can also be found in cruciferous vegetables, exerts multiple biological effects, such as anti-microbial [13,14], anti-inflammatory [15,16], anti-angiogenic [17], and anti-cancer activities. It inhibits the proliferation of human bladder cancer cells [18] and suppresses the cell metastasis of colorectal adenocarcinoma [19], bladder cancer [20], and hepatoma [21] cells. It also induce apoptotic cell death in bladder cancer cells [18], cervical cancer cells [22], hepatoma cells [20], colorectal cancer cells [23], and human cisplatin-resistant oral cancer cells [24]. Moreover, in in vivo studies, AITC has been shown to reduce liver fibrosis by regulating Kupffer cell activation [25], and to facilitate lipid accumulation and inflammation via the Sirt1/AMPK and NF-κB signaling pathways in rats with non-alcoholic fatty liver disease [16]. AITC also acts as a hepatoprotective agent in the acetaminophen-induced liver injury of rats by inducing NRF2 activation [26].

Recently, the combination therapy of radiation and AITC induces a replication-associated DNA damage response in non-small cell lung cancer (NSCLC) cells [27]. More interesting is that AITC has been shown to induce cell apoptosis in human brain malignant glioma GBM8401 cells through a mitochondria-dependent pathway [28]. However, there is no available information to confirm whether AITC suppresses brain tumor cells in animal models in vivo. Therefore, in the present study, we investigated the effects of AITC on tumor growth inhibition in human glioblastoma GBM8401/*luc2* cell xenograft mice. Our results indicate that AITC significantly reduced tumor growth in GBM8401/*luc2* cell xenograft nude mice in vivo. These findings confirm the results of previous in vitro studies, and provide more information on AITC activities in vivo. Thus, these observations may provide further support for the consideration of AITC for future use in the treatment of glioblastoma patients.

## 2. Results

### 2.1. AITC Affected Cell Viability and Apoptosis in GBM8401/luc2 Cells

**Figure 1:**
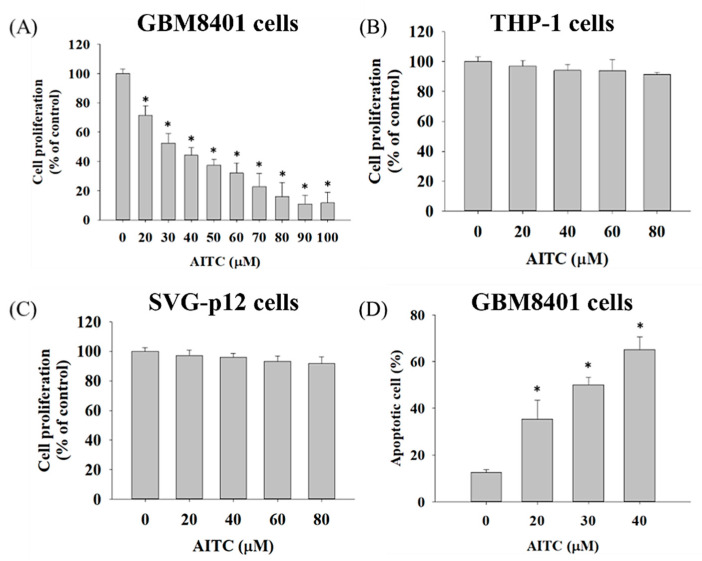
AITC decreased cell viability and induced cell apoptosis in GBM8401/*luc2* cells. Cells (1 × 10^4^ cells/well) were maintained in 96-well, and were treated with 0, 20, 30, 40, 50, 60, 70, 80, 90, and 100 μM of AITC for 48 h. After treatment, (**A**) GBM8401/*luc2* cells, (**B**) THP-1, and (**C**) SVG-p12 cells were collected for measuring cell viability and (**D**) apoptosis of GBM8401/*luc2* cells described in the Materials and Methods. (* Significantly different at *p* < 0.05 vs. the control group). Each treatment was replicated 3 times with 12 wells per group.

First of all, we aimed to identify the toxicity effect of AITC on GBM cells. After the GBM8401/*luc2* cells had been treated with various concentrations of AITC for 48 h, we measured cell viability and apoptosis; the results are presented in Figure 1. Cell viability indicated that AITC at 20–100 µM decreased the percentage of viable cells (cell viability) from 28–88% as compared to control cells (0 µM), and these effects were dose-dependent (Figure 1A). THP-1, represented as a monocyte, was used to confirm that there was no obvious systemic toxicity found in normal monocytes (Figure 1B). In addition, we also identified that AITC may induce normal neuron toxicity by MTT assay. As indicated in Figure 1C, the AITC treatment may not induce cytotoxicity in SVG-p12 cells (astroglia cells, specifically, remain in the brain). After the cytotoxicity of AITC in GBM401/*luc2* cells had been indicated, we then further identified that AITC-induced cell death is associated with apoptosis induction in GBM401/*luc2* cells. Furthermore, the results of the Annexin V/PI staining assay indicate that AITC significantly increased the occurrence of apoptotic cell death (cell apoptosis) from 35–65% compared to the control (Figure 1D). These results indicate that AITC may induce cytotoxicity in GBM401/*luc2* cells, and is associated with apoptosis mechanism induction.

### 2.2. AITC Affected Apoptotic-Cell-Death-Associated Protein Expression in GBM8401/luc2 Cells

**Figure 2:**
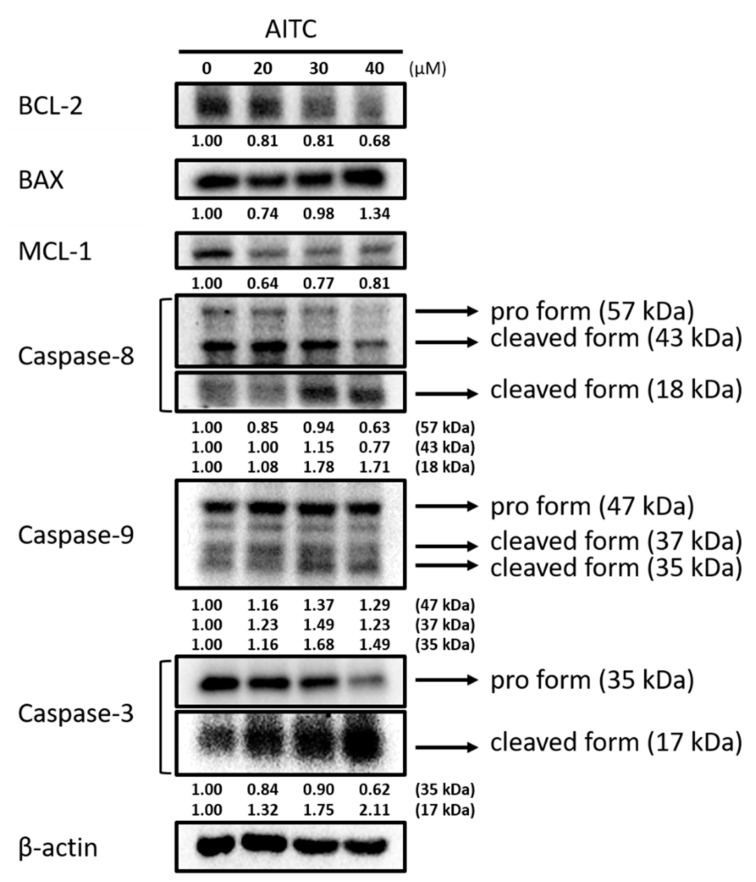
AITC affected apoptosis-associated protein expression in GBM8401/*luc2* cells. Cells (1.6 × 10^6^ cells) were treated with 0, 20, 30, and 40 μM of AITC for 48 h. Cells were harvested for examining apoptosis-associated protein expression evaluation by Western blotting as described in the Materials and Methods. Each treatment was replicated 3 times with 3 independent samples.

Cells were treated with various concentrations of AITC for 48 h, and were examined for apoptotic-cell-death-associated protein expression by Western blotting; the results are presented in Figure 2. The results indicate that AITC increased pro-apoptotic protein expression at 40 µM and decreased 20–40% of the anti-apoptotic protein BCL-2 and MCL-1 expression at 20–40 µM in GBM8401/*luc2* cells. The inhibition rate of BCL-2 and MCL-1 protein expression varied from 20–40% depending on AITC dose treatment. Furthermore, AITC at 20–40 µM increased the levels of cleaved caspase-3, -8, and -9 in GBM8401/*luc2* cells. The induction rates of apoptotic markers by AITC were 1.5–2 times greater compared to nontreated controls. These results suggest that AITC may induce apoptosis-related protein expression, but suppress anti-apoptosis-related protein expression.

### 2.3. Tumor Inhibition Effect of AITC on Glioblastoma Animal Model

**Figure 3:**
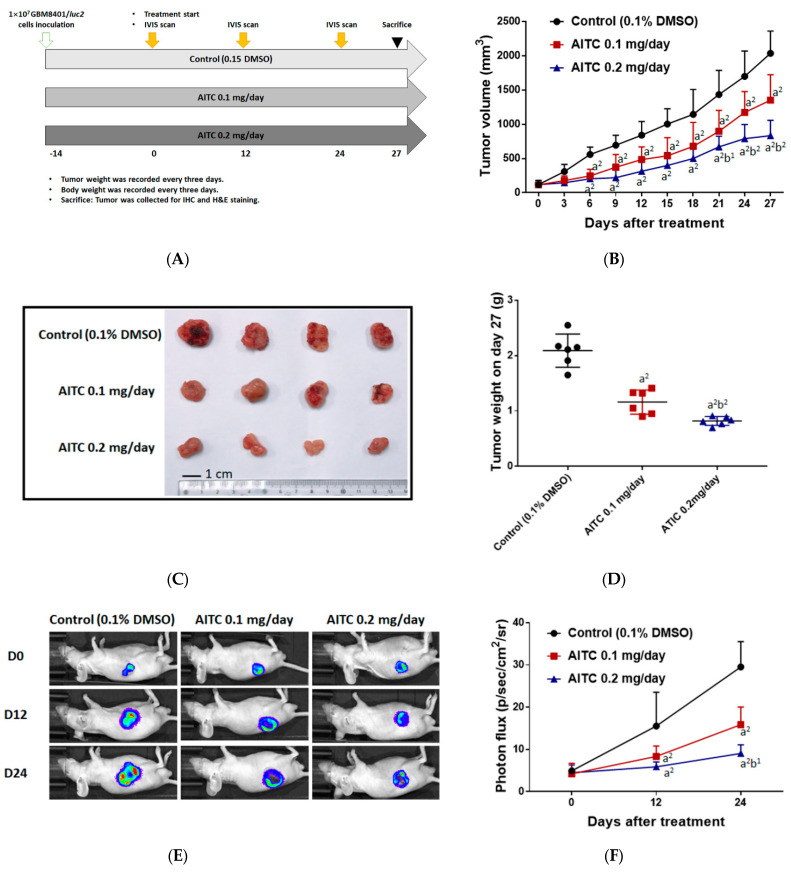
AITC effectively suppressed glioblastoma growth. (**A**) The animal experiment flow chart. (**B**) Tumor volume of control and AITC-treated groups (0.1 and 0.2 mg/day) from day 0 to day 27. (**C**) The representative tumor images from each group. (**D**) The tumor weights of mice on day 27. (**E**) The representative BLI results from each group at different time points. (**F**) Quantification results of luc2 signal intensity of tumors. (a^2^ *p* < 0.01 vs. control; b^1^ *p* < 0.05, b^2^ *p* < 0.01 vs. AITC 0.1 mg/day). Each treatment was replicated 2 times with 4 mice per group.

In order to identify the tumor inhibition effect of AITC, we established a GBM8401/*luc2*-cell-bearing animal model (Figure 3A). Mice were separated into three groups, comprising one control (0.1% DMSO) and two treatment groups (0.1 mg/day of AITC and 0.2 mg/day of AITC), once tumor volumes had reached 100–120 mm^3^. Tumor volume was also recorded every 3 days during treatment and mice were sacrificed on day 27 for further experiments. As illustrated in Figure 3B, the smallest tumor size and the slowest progression pattern were both found in the 0.2 mg/day of AITC group, indicating the tumor inhibition effect of AITC. The tumor inhibition rate on day 27 was around 75% in AITC 0.2 mg/day group as compared to the control group. Moreover, tumors extracted from individual mice on day 27 were imaged and evaluated in terms of their weight. As shown in Figure 3C,D, the tumors were smaller and lighter in the 0.2 mg/day of AITC group than the other two groups. The weights of the control tumors were 2–3 times greater compared to AITC 0.2 mg/day group. To investigate whether tumor growth inhibition was mainly caused by the living cell loss within the tumor region, we established the luc2 reporter gene system in GBM8401 cells. Mice were subjected to an IVIS scan on days 0, 12, and 24 after treatment to identify living cell signals within the tumor. As presented in Figure 3E,F, the photon signal was markedly reduced in the 0.2 mg/day of AITC group by at least half of a percent signal, which indicates that the loss of living cells increased after AITC treatment. Taken together, AITC may suppress glioblastoma tumor growth in a dose-dependent manner, based on our results for tumor size, tumor weight, and living cell signaling within the tumor region.

### 2.4. AITC Treatment May Not Cause Tissue Damage and Body Weight Loss

**Figure 4:**
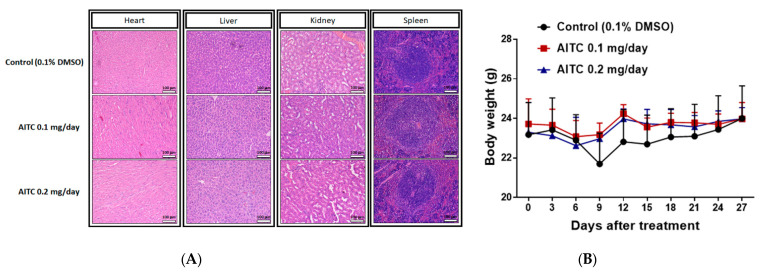
The examinations of AITC-induced toxicity from xenograft GBM8401/*luc2*-cell-bearing mice. After treatment, tissues of the heart, liver, kidney, and spleen were isolated from each mouse and used in H&E staining for further examination of the pathology under the microscope at 100 times magnification, as described in the Materials and Methods (**A**). Mouse body weights of control and AITC-treated groups from day 0 to day 27 are recorded and quantified (**B**). Each section was imaged with 3 independent fields. (Scale bar = 100 μm, with 200× magnification).

To investigate whether AITC treatment may induce tissue toxicity, we analyzed animal tissue pathology by H&E staining. As shown in Figure 4A, no apparent tissue pathology alteration, including heart, liver, kidney, and spleen tissues, was found in mice treated with 0.1 and 0.2 mg/day of AITC. The pathology was also interpreted by a veterinarian with pathological interpretation experience of more than 5 years. As illustrated in Table 1, extramedullary hematopoiesis with mildly increased megakaryocytes was noted in the liver and red pulp of the spleen in CT mice. However, these changes were minimal in 0.2 mg/day of AITC-treated mice. In addition, we also recorded the body weight of mice during the treatment period. In Figure 4B, no significant body weight changes were found in any of the groups of mice, indicating no apparent toxicity in AITC-treated groups.

### 2.5. AITC Affected Tumor Progression- and Apoptosis-Related Protein Expression

**Figure 5:**
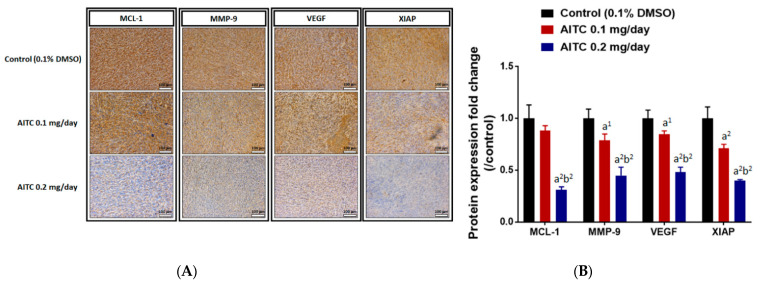
AITC suppressed the expression of tumor-progression-related proteins. Tumors were isolated from each group and further examined by IHC staining. The IHC staining images of MCL-1, MMP-9, VEGF, XIAP on the tumor were observed by microscope at 100 times magnification (**A**). The quantification data of MCL-1, MMP-9, VEGF, XIAP expressions is presented (**B**). (a^1^ *p* < 0.05, a^2^ *p* < 0.01 vs. control; b^2^ *p* < 0.01 vs. AITC 0.1 mg/day). Each section was imaged with 3 independent fields and quantified. (Scale bar = 100 μm, with 200× magnification).

To identify the underlying mechanism of tumor growth inhibition, we further investigated the alteration of tumor-progression-related protein expression, including MCL-1, XIAP, MMP-9, and VEGF after AITC treatment by IHC staining of tumor tissues. As illustrated in Figure 5A,B, AITC effectively suppressed protein expressions of MCL-1, MMP-9, VEGF, and XIAP in the tumor area. The suppression percentage of theses oncogenes in ATIC 0.1 mg/day is around 20% as compared to control group. The suppression effect in ATIC 0.2 mg/day is around 40–60% as compared to control group. The positive staining results was found to be decreased by AITC in a dose-dependent manner.

**Figure 6:**
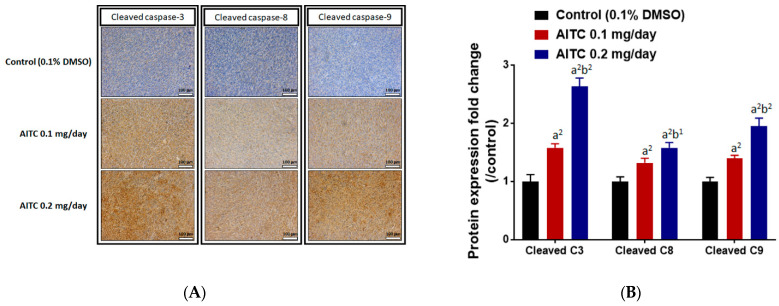
AITC induced the expressions of apoptosis-related proteins. Tumors were isolated from each group and further examined by IHC staining. The IHC staining images of cleaved caspase-3, -8, and -9 on the mouse tumors were observed by microscope at 100 times magnification (**A**). The quantification data of cleaved caspase-3, -8, and -9 expressions are presented (**B**). (a^2^ *p* < 0.01 vs. control; b^1^ *p* < 0.05, b^2^ *p* < 0.01 vs. AITC 0.1 mg/day). Each section was imaged with 3 independent fields and quantified. (Scale bar = 100 μm, with 200× magnification).

We also investigated whether AITC triggered apoptosis effects in glioblastoma. IHC protein staining results from the tumor indicated that the induction of cleaved caspase-3, -8, and -9 occurred in the AITC treatment tumor (Figure 6A). In Figure 6B, the apoptosis protein expressions were increased in a dose-dependent manner after AITC 27 days’ treatment (brown color pattern that represented a positive staining result was increased). The induction rates of these apoptosis genes were 1.5–2.5 times higher in the AITC-treated group as compared to the control group. In conclusion, AITC may suppress tumor-progression-related protein expression, but induce caspase-dependent protein expression.

## 3. Discussion

Brain tumors are one of the main causes of death in the human population, and the current standard treatment fails to cure most patients with brain tumors [1,2]; thus, the median survival is about 12–18 months [4,5]. Currently, most GBM patients are treated with TMZ, although GBM typically recurs after two-year treatment [29]; furthermore, this treatment has significant long-term debilitating side effects. Therefore, finding new drugs with minor side effects and substances that can prevent tumor growth is critical. Numerous studies have shown that AITC exhibits anti-cancer activities in many human cancer cell lines. However, there are no reports to show that AITC suppresses human glioblastoma GBM8401 cells in vivo. In vivo animal models represent a more desirable approach for studying the anti-tumor activity of chemical and cancer diseases as a whole. The simple protocol of the subcutaneous xenograft model has been used extensively in cancer research, especially regarding the activity of anti-cancer drugs [30,31].

The use of immunodeficient animals (athymic mice or rats) for in vivo experiments to measure the efficiency of test chemicals, including natural products, for anti-tumor activities based on patient-derived tumor xenografts has provided new insights in many clinical fields. Therefore, thymic BALB/c nude mice were selected for the cancer-cell-generated xenograft mice model in vivo [32]. Our earlier studies have established GBM8401/*luc2* cell xenograft tumors in athymic BALB/c nude mice to investigate whether ITC-associated compounds (BITC and PEITC) retard tumors in vivo [33,34]. In the present study, we aimed to determine whether AITC is a suitable compound for in vivo studies. Thus, in the primary experiments, we measured the cytotoxicity effects (cell viability and apoptosis) of AITC on GBM8401/*luc2* cells in vitro, and the results are shown in Figure 1A,B. In normal human bladder epithelial cells, the IC_50_ value of AITC is approximately 10 times higher than that in human bladder cancer cells [17]. Therefore, in our study, we did not need to spend further time and cost in conducting a normal cell experiment.

Our results indicate that AITC significantly reduced the total percentage of cell viability and induced cell apoptosis in GBM8401/*luc2* cells (Figure 1). To ascertain whether cell apoptosis involved changes in apoptosis-associated proteins, we used Western blotting to investigate the effects of AITC on apoptosis-associated protein expression; the results are presented in Figure 2. These results indicate that AITC increased pro-apoptotic protein expression at 40 µM, but decreased anti-apoptotic protein BCL-2 and MCL-1 expression at 20–40 µM, in GBM8401/*luc2* cells. Furthermore, AITC at 20–40 µM increased the levels of cleaved caspase-3, -8, and -9 in GBM8401/*luc2* cells, which means that AITC induced apoptosis-related cell death via caspase-dependent pathways in GBM8401/*luc2* cells. These results indicate that AITC may induce cytotoxicity in GBM8401/*luc2* cells via the activation of caspase-dependent apoptosis mechanisms. This is in agreement with other reports showing that AITC induces human cancer cell apoptosis via caspase-dependent pathways [24,35].

Based on the observations from in vitro studies, we examined the effects of AITC on tumor growth in nude mice (Figure 3A), and our results indicate that AITC at both doses (0.1 and 0.2 mg/day) significantly inhibited the tumor volume and weight in GBM8401/*luc2*-cell-generated xenograft nude mice in vivo (Figure 3B–D). Comparisons of tumor growth between the treatment groups revealed that the higher dose (0.2 mg/day) of AITC had a higher inhibitory effect than that of the low dose (0.1 mg/day) (Figure 3B–D). During the treatment of AITC, we also used luciferase-bearing cells to monitor tumor growth, and these results are shown in Figure 3E,F. Evaluating the BLI results for each group of mice, relatively weak signals were found in the AITC-treated groups compared to the control group (Figure 3E). The total photon flux from the control group was two to four times higher than that of AITC-treated mice, and the higher dose of AITC had a lower photon flux than that of the lower dose (Figure 3F). Therefore, AITC treatment may reduce the living cell population within the tumor area. These findings agree with the tumor size and weight results (Figure 3B–D). The inhibition of growth observed in GBM8401/*luc2* cell xenograft mice tumors provides evidence that AITC was active in these tumor models. Overall, AITC significantly induced apoptotic cell death in vitro and suppressed tumor growth in vivo.

The anti-tumor and toxic effects of AITC treatments should be considered. Some therapeutic drugs possess toxic side effects such as weight loss, cytotoxicity to normal cells at high treatment doses, and organ injuries. These toxicity characters may be associated with higher morbidity and lower response rate, resistance, and recurrence, resulting in poorer survival in clinical patients. In the present study, during the treatments of AITC on GBM8401/*luc2* cell xenograft mice, the body weights of the mice were individually recorded for each group. The results show that there were no differences between the AITC-treated and control groups (Figure 4B). The body weight index and tissue H&E staining were utilized to evaluate the pathology of experimental animals and whether or not they were affected by test agents. After analyzing hearts, livers, spleens, and kidneys from individual mice using H&E staining, there were no significant pathologic differences found between AITC-treated and control groups (Figure 4A).

A tumor-inhibiting agent may show the potential to block multiple hallmark tumor capabilities [36], including triggering cell toxicity through the induction of cancer cell apoptosis without causing normal cellular toxicity. Simultaneously, it may alter protein expressions or block multiple cancer-associated pathways. Herein, to further confirm AITC activity involved in tumor growth retardation in GBM8401/*luc2* cell xenograft mice, we used tumor samples from individual mice to investigate apoptosis-associated protein expression via an immunostaining assay on tumor sections. The results indicate that AITC decreased the levels of myeloid cell lymphoma-1 (MCL-1), matrix metalloproteinase-9 (MMP-9), vascular endothelial growth factor (VEGF), and X-linked inhibitor of apoptosis (XIAP) (Figure 5), and increased the expression of caspase-3, -8, and -9 in tumor sections (Figure 5). Moreover, these findings revealed that AITC significantly decreased the expression of anti-apoptotic proteins such as MCL-1 and XIAP compared to the control groups (*p* < 0.001). MCL-1, a unique component among anti-apoptotic Bcl-2 proteins, is an early response gene with rapid induction and turnover rates [37], and plays a unique and fundamental role in regulating apoptosis [38]. If the MCL-1 expression is inhibited, apoptosis induction could develop in certain malignancies [39,40]. XIAP prevents apoptosis through the direct inhibition of effector caspases (caspase-3, -6, -7, and -9) [41]. XIAP plays an anti-apoptotic function, and it also inhibits autophagy [42]. Thus, more attention has thus far been focused on XIAP suppression for developing new strategies to prevent and/or treat cancer [43]. Similar findings regarding the impact of AITC on other cancer cells in vivo from previous reports indicate that AITC inhibits the growth of human prostate cancer PC-3 cell xenografts in vivo via both inducing apoptosis and reducing mitotic activity [44].

Vascular endothelial growth factor (VEGF) plays a critical role in the formation of vessels and in physiologic vascular homeostasis in diverse cells and tissues; it is also needed for tumor growth and metastasis and inhibits endothelial cell apoptosis [45,46]. VEGF has been used in multiple clinical trials as an essential target for solid tumor treatment in multiple disease settings. Proteins in the MMP family have been shown to play crucial roles in cancer cell adhesion, migration, and invasion [47,48]. MMP-9 and VEGF are thought to be involved in tumor progression and metastasis. Thus, we further investigated whether or not AITC also affects MMP-9 and VEGF, and our results indicate that AITC suppressed both of these proteins (Figure 5A,B). Increased expression of MMP-9 was associated with poor prognosis, and its downregulation is one of the strategies used to improve the outcome of ovarian cancer [49]. In addition, blocking MMP-9 expression by anti-mRNA led to the inhibition of cancer cell invasion and angiogenesis in human glioma cells [50]. Our results provide solid evidence that AITC can significantly reduce VEGF and MMP-9 in the tumor samples of GBM8401/*luc2* cell xenograft mice.

Caspases mediate cell apoptosis after stimulation; they can be divided into initiators of apoptosis, such as caspase-2, -8, -9, -10, and -12, and effectors of apoptosis, such as caspase-3, -6, and -7 [51]. Caspase-dependent pathways include the death receptor caspase-8- and the mitochondrial caspase-9-dependent pathways. Therefore, we examined the effects of AITC on the expressions of cleaved caspase -3, -8, and -9 in tumor tissues from GBM8401/*luc2* cell xenograft mice. Our results indicate that AITC significantly increased the levels of cleaved caspase -3, -8, and -9 (Figure 6A,B) when compared to the control group. Caspase-3 plays a critical apoptosis effector, and is activated by two different pathways, the upstream initiating subsystem (caspase-8) and the caspase-9-dependent pathway, and finally leads to cell apoptosis [52,53]. The agents can induce tumor cell apoptosis during the therapeutic process to reduce tumor size [53].

It was previously suggested that AITC reduces tumor growth of Ehrlich ascites tumor (EAT) cells in vivo through both antiangiogenic and pro-apoptotic mechanisms [54].

**Figure 7:**
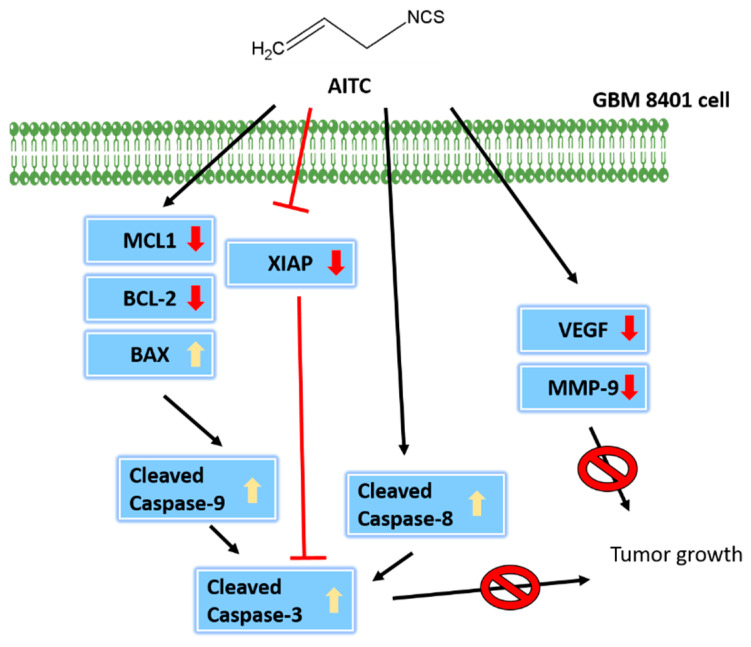
The mechanisms involved in AITC-driven inhibition of tumor growth.

In conclusion, GBM8401/*luc2* cell xenograft tumor growth in athymic BALB/c nude mouse models for human glioma cancer was successfully developed, which could be useful in studying AITC-retarded tumor growth in vivo. The findings of this research indicate that AITC treatment significantly decreases the tumor volume and weight, and did not considerably affect the organs, namely the heart, liver, spleen, and kidney, based on tissues H&E staining, indicating no significant systemic toxicity to the mice. Furthermore, tumor tissues were stained with tumor progression- and anti-apoptotic-associated antibodies, indicating that AITC induced cell apoptosis for retarding tumor growth. Overall, the possible signaling pathways used by AITC to decrease tumor size through the inhibition of caspase-3, -8, and -9 in GBM8401/*luc2* cells are presented in Figure 7.

## 4. Materials and Methods

### 4.1. Test Chemicals, Reagents, Culture Medium, and Antibodies

Allyl isothiocyanate (AITC) and dimethyl sulfoxide (DMSO) were purchased from Sigma Chemical Co. (St. Louis, MI, USA). AITC was dissolved in DMSO as 150 mg/mL stock. Roswell Park Memorial Institute (RPMI) 1640 medium, fetal bovine serum (FBS), L-glutamine, and penicillin/streptomycin (PS) were purchased from Gibco/Life Technologies (Carlsbad, CA, USA), respectively. D-luciferin, pGL4.50 luciferase reporter (pGL4.50[luc2/CMV]) vector, and hygromycin B were obtained from Promega (Madison, WI, USA) and Santa Cruz Biotechnology (Santa Cruz, CA, USA), respectively. JetPEI™ transfection reagent was obtained from Polyplus Transfection (Illkirch, Bas-Rhin, France).

### 4.2. Culture of Human Glioblastoma GBM8401 Cells

Human glioblastoma GBM8401 cell line was obtained from the Food Industry Research and Development Institute (Hsinchu, Taiwan). GBM8401 cells were placed in a 10-cm dish with RPMI 1640 supplemented with 10% FBS and penicillin (100 U/mL)/streptomycin (100 µg/mL) at 37 °C under a humidified 5% CO_2_ atmosphere of the incubator as described previously [33,55].

### 4.3. Cell Transfection and Stable Clone Selection

Plasmid transfection and stable clone selection protocol were described previously [56,57]. In brief, GBM8401 cells in the plates were added with pGL4.50 luciferase reporter (pGL4.50[luc2/CMV]) plasmid and JetPEI™ transfection reagents mixture overnight. A 200 μg/mL hygromycin B was used for screening and maintaining the luc2-expressing cells in GBM8401 cells. The Luc2 signaling was acquired by IVIS 200 Imaging System (Xenogen, Alameda, CA, USA). Cells with stable expression of luc2 reporter gene were obtained and defined as GBM8401/*luc2* for further experiments used in this study.

### 4.4. Cell Viability and Apoptosis Assays

GBM8401/*luc2* cells (1 × 10^4^ cells/well) were planted in 96-well plates with RPMI 1640 and were incubated with various concentrations of AITC (0, 20, 30, 40, 50, 60, 70, 80, 90, and 100 μM) for 48 h. For cell viability assay, cells were added 10 μL MTT (5 mg/mL) for 4 h, and then added to 100 μL 10% SDS/0.01 M HCl solution overnight. After incubation, the absorbance was read with an ELISA reader at 595 nm as described previously [58]. For cell apoptosis assays, cells were incubated with AITC (0, 20, 30, and 40 μM) for 48 h and were collected, re-suspended in 100 μL Annexin V binding buffer, and incubated with Annexin V-FITC/PI in the dark. The cells were subsequently analyzed for cell apoptosis by flow cytometer (BD Biosciences, FACSCalibur, San Jose, CA, USA) as described previously [33,55]. All cells from each treatment were performed in triplicate and the experiment was repeated three times for statistical analysis.

### 4.5. Western Blot Assays

GBM8401/*luc2* cells (1.6 × 10^6^ cells) in 10-cm dish were treated with 0, 20, 30, and 40 μM of AITC for 48 h. The cells from each treatment were harvested, lysed, and, using a Bio-Rad Protein Assay Kit (Bio-Rad Laboratories, Inc., Hercules, CA, USA), the total proteins were determined as previously described [33,55]. A sample of 30 μg protein from each treatment was dissolved using 10% sodium dodecyl sulfate-polyacrylamide gel (SDS page) electrophoresis and transferred to an Immobilon-P Transfer Membrane (Merck Millipore). Each blot was soaked in a blocking buffer (5% nonfat powdered milk and 0.05% Tween-20 in 1X Tris-buffered saline at pH 7.6) for 1 h at room temperature. All blots were individually incubated with primary monoclonal antibodies in the blocking buffer at 4 °C overnight, including BAX (#2772, dilution 1:1000), BCL-2 (#2870, dilution 1:1000), caspase-9 (#9508, dilution 1:1000), caspase-8 (#9746, dilution 1:1000), and caspase-3 (#9662, dilution 1:1000) were purchased from Cell Signaling Technology, Inc., and MCL-1 (M8434, dilution 1:8000) was purchased from Sigma Chemical Co. After washing, the blots were probed with Anti-mouse IgG, HRP-linked antibody (#7076, dilution 1:10,000), or Anti-rabbit IgG, HRP-linked antibody (#7074, dilution 1:10,000) (Cell Signaling Technology, Inc., Danvers, MA, USA) as previously described [33,55]. The anti-β-actin antibody was used to ensure equal protein loading from each sample. Quantitative analysis of each immunoreactive blot as previously described [33,55].

### 4.6. Development of Glioblastoma-Xenograft-Bearing Mice and AITC Treatment

Eighteen 6-week-old nude mice (CAnN.Cg-Fo*xn1nu*/*CrlNarl mice*) weighing 20–22 g were purchased from the National Laboratory Animal Center, Taipei, Taiwan. The experiment was approved by the Institutional Animal Care and Use Committee (IACUC) of China Medical University (ID number 2020-248). For each mouse, xenografts were inoculated into the right flank, comprising of 1 × 10^7^ GBM8401/*luc2* cells in 100 μL PBS with 30% Matrigel, and allowed to grow for 14 days. When the tumor reached 100–120 mm^3^, mice were separated into three groups (n = 6/per group). Control mice were treated daily with 0.1% DMSO in 100 μL phosphate-buffered saline (PBS) by gavage. Treatment groups were divided as 0.1 mg/day and 0.2 mg/day of AITC (in 100 μL of PBS) groups, with AITC administered by gavage. Mice were subjected to an IVIS scan on day 0, 12, and 24, and sacrificed on day 27 after treatment (Figure 3A). Mouse tumor and body weight were recorded every 3 days. Tumor volume was calculated following the formula V = length × width^2^ × 0.523 [59].

### 4.7. Animal IVIS Scan

Luc2 signals representative of living cells within tumors were acquired by IVIS^®^ SpectrumCT In Vivo Imaging System. Mice from each group were anesthetized with 1–2% isoflurane during scanning. The quantification of the Luc2 signal was performed by XENOGEN Living Image version 2.20.1 software. The unit of Luc2 signal from the region of interest was defined as photon/sec/cm^2^/sr [60].

### 4.8. Hematoxylin and Eosin (H&E) and Immunohistochemistry (IHC) Staining

Tumor, heart, spleen, kidney, and liver samples were extracted from mice on day 27. Heart, spleen, kidney, and liver tissues were analyzed via H&E staining to identify whether treatment induced tissue toxicity. H&E staining was performed at Bio-Check Laboratories Ltd. (New Taipei City, Taiwan) as described by Liu et al. [61]. The relative severity of histopathologic changes was recorded semi-quantitatively using Shackelford’s (2002) four-scale method with at least twenty 200× fields each section. Briefly, the severity was graded as: 0, regular tissue or minimal; 1, mild; 2, moderate; 3, moderate/severe; 4, severe/high. The scoring examination was performed by a pathologist (C-C Cheng), who was blinded to the mouse treatment assignment. Tumor tissues were performed for identifying the expressions of MCL-1, MMP-9, VEGF, XIAP, cleaved caspase-3, -8, and -9 proteins by IHC staining according to the instruction of EMD Millipore’s IHC Select^®^ Kit (EMD Millipore, Billerica, MA, USA) and described in our previous study [62]. Staining slides were then photographed by Nikon microscope at 100× magnification (Nikon ECLIPSE Ti-U, Minato City, Tokyo, Japan). Positive staining of each specific protein in IHC stain was quantified by ImageJ software version 1.50 (National Institutes of Health, Bethesda, MA, USA) using Immunohistochemistry (IHC) Image Analysis Toolbox. Primary antibodies were purchased from various companies, including cleaved caspase-3 (E-AB-30004, Elabscience, Houston, TX, USA), cleaved caspase-8 (E-AB-22107, Elabscience), cleaved caspase-9 (10380-1-AP, Proteintech, Rosemont, IL, USA), MCL-1 (E-AB-33430, Elabscience), MMP-9 (AG0549, Proteintech), VEGF (E-AB-70013, Elabscience), and XIAP (E-AB-61374, Elabscience).

### 4.9. Statistical Analysis

One-way ANOVA was performed using GraphPad Prism 7.0 version (San Diego, CA, USA). A *p*-value smaller than 0.05 was considered to represent a significant difference between control and treatment groups. Data are presented as mean ± standard deviation.

## Figures and Tables

**Table 1:** The alteration of pathology was defined as the following 4 severity scores: 0—regular tissue, 1—mild changes, 2—moderate changes, 3—significant changes. The interpretation was performed by a veterinarian with pathological interpretation experience of more than 5 years. Each group of mice was examined with two different slices (slice A and B) for 3–4 regions. (-, no sample).

Slide	Section	Score of the Region			
		R1	R2	R3	R4
CT_A CT_B	Heart	0	0	-	-
		0	0	-	-
AITC 0.1_A AITC 0.1_B	Heart	0–1	0–1	-	-
		0	0	-	-
AITC 0.2_A AITC 0.2_B	Heart	0	0	-	-
		0	0	-	-
CT_A CT_B	Liver	0	0	1	0
		0	0	1	0
AITC 0.1_A AITC 0.1_B	Liver	0	0–1	0	0–1
		0	0–1	0	0–1
AITC 0.2_A AITC 0.2_B	Liver	0	0–1	0	0–1
		0	0–1	0	0–1
CT_A CT_B	Kidney	0	0	0–1	0
		0	0	0–1	0
AITC 0.1_A AITC 0.1_B	Kidney	0	0	0–1	0–1
		0	0	0–1	0–1
AITC 0.2_A AITC 0.2_B	Kidney	0	0	0–1	0–1
		0	0	0–1	0–1
CT_A CT_B	Spleen	1	1	1	-
		1	1	1	-
AITC 0.1_A AITC 0.1_B	Spleen	0	0	0	-
		0	0	0	-
AITC 0.2_A AITC 0.2_B	Spleen	0	0	0	-
		0	0	0	-

## Data Availability

Data available on request from corresponding author.

## References

[1] Sturm D., Bender S., Jones D.T., Lichter P., Grill J., Becher O., Hawkins C., Majewski J., Jones C., Costello J.F. (2014). Paediatric and adult glioblastoma: Multiform (epi) genomic culprits emerge. Nat. Rev. Cancer.

[2] Louis D.N., Ohgaki H., Wiestler O.D., Cavenee W.K., Burger P.C., Jouvet A., Scheithauer B.W., Kleihues P. (2007). The 2007 WHO classification of tumours of the central nervous system. Acta Neuropathol..

[3] Brown J.M. (2014). Vasculogenesis: A crucial player in the resistance of solid tumours to radiotherapy. Br. J. Radiol..

[4] Stupp R., Hegi M.E., Mason W.P., van den Bent M.J., Taphoorn M.J., Janzer R.C., Ludwin S.K., Allgeier A., Fisher B., Belanger K. (2009). Effects of radiotherapy with concomitant and adjuvant temozolomide versus radiotherapy alone on survival in glioblastoma in a randomised phase III study: 5-year analysis of the EORTC-NCIC trial. Lancet Oncol..

[5] Stupp R., Mason W.P., van den Bent M.J., Weller M., Fisher B., Taphoorn M.J., Belanger K., Brandes A.A., Marosi C., Bogdahn U. (2005). Radiotherapy plus concomitant and adjuvant temozolomide for glioblastoma. N. Engl. J. Med..

[6] Wang L., Wei Q., Wang L.E., Aldape K.D., Cao Y., Okcu M.F., Hess K.R., El-Zein R., Gilbert M.R., Woo S.Y. (2006). Survival prediction in patients with glioblastoma multiforme by human telomerase genetic variation. J. Clin. Oncol..

[7] Yamanaka R., Hayano A., Kanayama T. (2018). Radiation-induced gliomas: A comprehensive review and meta-analysis. Neurosurg. Rev..

[8] Chikara S., Nagaprashantha L.D., Singhal J., Horne D., Awasthi S., Singhal S.S. (2018). Oxidative stress and dietary phytochemicals: Role in cancer chemoprevention and treatment. Cancer Lett..

[9] Lee H.-P., Chen P.-C., Wang S.-W., Fong Y.-C., Tsai C.-H., Tsai F.-J., Chung J.-G., Huang C.-Y., Yang J.-S., Hsu Y.-M. (2019). Plumbagin suppresses endothelial progenitor cell-related angiogenesis in vitro and in vivo. J. Funct. Foods.

[10] Tafrihi M., Nakhaei Sistani R. (2017). E-Cadherin/β-Catenin Complex: A Target for Anticancer and Antimetastasis Plants/Plant-derived Compounds. Nutr. Cancer.

[11] Mitsiogianni M., Mantso T., Trafalis D.T., Vasantha Rupasinghe H.P., Zoumpourlis V., Franco R., Botaitis S., Pappa A., Panayiotidis M.I. (2020). Allyl isothiocyanate regulates lysine acetylation and methylation marks in an experimental model of malignant melanoma. Eur. J. Nutr..

[12] Vanduchova A., Anzenbacher P., Anzenbacherova E. (2019). Isothiocyanate from Broccoli, Sulforaphane, and Its Properties. J. Med. Food.

[13] Tsai S.C., Huang W.W., Huang W.C., Lu C.C., Chiang J.H., Peng S.F., Chung J.G., Lin Y.H., Hsu Y.M., Amagaya S. (2012). ERK-modulated intrinsic signaling and G(2)/M phase arrest contribute to the induction of apoptotic death by allyl isothiocyanate in MDA-MB-468 human breast adenocarcinoma cells. Int. J. Oncol..

[14] Li Y., Liu Y., Zhang Z., Cao Y., Li J., Luo L. (2020). Allyl Isothiocyanate (AITC) Triggered Toxicity and FsYvc1 (a STRPC Family Member) Responded Sense in Fusarium solani. Front. Microbiol..

[15] Liu P., Behray M., Wang Q., Wang W., Zhou Z., Chao Y., Bao Y. (2018). Anti-cancer activities of allyl isothiocyanate and its conjugated silicon quantum dots. Sci. Rep..

[16] Li C.X., Gao J.G., Wan X.Y., Chen Y., Xu C.F., Feng Z.M., Zeng H., Lin Y.M., Ma H., Xu P. (2019). Allyl isothiocyanate ameliorates lipid accumulation and inflammation in nonalcoholic fatty liver disease via the Sirt1/AMPK and NF-κB signaling pathways. World J. Gastroenterol..

[17] Zhang Y. (2010). Allyl isothiocyanate as a cancer chemopreventive phytochemical. Mol. Nutr. Food Res..

[18] Sávio A.L., da Silva G.N., Salvadori D.M. (2015). Inhibition of bladder cancer cell proliferation by allyl isothiocyanate (mustard essential oil). Mutat. Res..

[19] Lai K.C., Lu C.C., Tang Y.J., Chiang J.H., Kuo D.H., Chen F.A., Chen I.L., Yang J.S. (2014). Allyl isothiocyanate inhibits cell metastasis through suppression of the MAPK pathways in epidermal growth factor-stimulated HT29 human colorectal adenocarcinoma cells. Oncol. Rep..

[20] Hwang E.S., Lee H.J. (2006). Allyl isothiocyanate and its N-acetylcysteine conjugate suppress metastasis via inhibition of invasion, migration, and matrix metalloproteinase-2/-9 activities in SK-Hep 1 human hepatoma cells. Exp. Biol. Med..

[21] Thejass P., Kuttan G. (2007). Inhibition of endothelial cell differentiation and proinflammatory cytokine production during angiogenesis by allyl isothiocyanate and phenyl isothiocyanate. Integr. Cancer Ther..

[22] Qin G., Li P., Xue Z. (2018). Effect of allyl isothiocyanate on the viability and apoptosis of the human cervical cancer HeLa cell line in vitro. Oncol. Lett..

[23] Chiang J.H., Tsai F.J., Hsu Y.M., Yin M.C., Chiu H.Y., Yang J.S. (2020). Sensitivity of allyl isothiocyanate to induce apoptosis via ER stress and the mitochondrial pathway upon ROS production in colorectal adenocarcinoma cells. Oncol. Rep..

[24] Chang P.Y., Tsai F.J., Bau D.T., Hsu Y.M., Yang J.S., Tu M.G., Chiang S.L. (2021). Potential effects of allyl isothiocyanate on inhibiting cellular proliferation and inducing apoptotic pathway in human cisplatin-resistant oral cancer cells. J. Formos. Med. Assoc..

[25] Kim J., Bang H., Ahn M., Choi Y., Kim G.O., Shin T. (2018). Allyl isothiocyanate reduces liver fibrosis by regulating Kupffer cell activation in rats. J. Vet. Med. Sci..

[26] Kim M.W., Kang J.H., Jung H.J., Park S.Y., Phan T.H.L., Namgung H., Seo S.Y., Yoon Y.S., Oh S.H. (2020). Allyl Isothiocyanate Protects Acetaminophen-Induced Liver Injury via NRF2 Activation by Decreasing Spontaneous Degradation in Hepatocyte. Nutrients.

[27] Tripathi K., Hussein U.K., Anupalli R., Barnett R., Bachaboina L., Scalici J., Rocconi R.P., Owen L.B., Piazza G.A., Palle K. (2015). Allyl isothiocyanate induces replication-associated DNA damage response in NSCLC cells and sensitizes to ionizing radiation. Oncotarget.

[28] Chen N.G., Chen K.T., Lu C.C., Lan Y.H., Lai C.H., Chung Y.T., Yang J.S., Lin Y.C. (2010). Allyl isothiocyanate triggers G2/M phase arrest and apoptosis in human brain malignant glioma GBM 8401 cells through a mitochondria-dependent pathway. Oncol. Rep..

[29] Adamson C., Kanu O.O., Mehta A.I., Di C., Lin N., Mattox A.K., Bigner D.D. (2009). Glioblastoma multiforme: A review of where we have been and where we are going. Expert Opin. Investig. Drugs.

[30] Hakkarainen T., Särkioja M., Lehenkari P., Miettinen S., Ylikomi T., Suuronen R., Desmond R.A., Kanerva A., Hemminki A. (2007). Human mesenchymal stem cells lack tumor tropism but enhance the antitumor activity of oncolytic adenoviruses in orthotopic lung and breast tumors. Hum. Gene Ther..

[31] Poplin E., Feng Y., Berlin J., Rothenberg M.L., Hochster H., Mitchell E., Alberts S., O’Dwyer P., Haller D., Catalano P. (2009). Phase III, randomized study of gemcitabine and oxaliplatin versus gemcitabine (fixed-dose rate infusion) compared with gemcitabine (30-minute infusion) in patients with pancreatic carcinoma E6201: A trial of the Eastern Cooperative Oncology Group. J. Clin. Oncol..

[32] Mattern J., Bak M., Hahn E.W., Volm M. (1988). Human tumor xenografts as model for drug testing. Cancer Metastasis Rev..

[33] Chou Y.C., Chang M.Y., Lee H.T., Shen C.C., Harnod T., Liang Y.J., Wu R.S., Lai K.C., Hsu F.T., Chung J.G. (2018). Phenethyl Isothiocyanate Inhibits In Vivo Growth of Xenograft Tumors of Human Glioblastoma Cells. Molecules.

[34] Ma Y.S., Lin J.J., Lin C.C., Lien J.C., Peng S.F., Fan M.J., Hsu F.T., Chung J.G. (2018). Benzyl isothiocyanate inhibits human brain glioblastoma multiforme GBM 8401 cell xenograft tumor in nude mice in vivo. Environ. Toxicol..

[35] Bo P., Lien J.C., Chen Y.Y., Yu F.S., Lu H.F., Yu C.S., Chou Y.C., Yu C.C., Chung J.G. (2016). Allyl Isothiocyanate Induces Cell Toxicity by Multiple Pathways in Human Breast Cancer Cells. Am. J. Chin. Med..

[36] Hanahan D., Weinberg R.A. (2000). The hallmarks of cancer. Cell.

[37] Yang T., Kozopas K.M., Craig R.W. (1995). The intracellular distribution and pattern of expression of Mcl-1 overlap with, but are not identical to, those of Bcl-2. J. Cell Biol..

[38] Nijhawan D., Fang M., Traer E., Zhong Q., Gao W., Du F., Wang X. (2003). Elimination of Mcl-1 is required for the initiation of apoptosis following ultraviolet irradiation. Genes Dev..

[39] Huang X., Wu Z., Mei Y., Wu M. (2013). XIAP inhibits autophagy via XIAP-Mdm2-p53 signalling. EMBO J..

[40] Podar K., Gouill S.L., Zhang J., Opferman J.T., Zorn E., Tai Y.T., Hideshima T., Amiot M., Chauhan D., Harousseau J.L. (2008). A pivotal role for Mcl-1 in Bortezomib-induced apoptosis. Oncogene.

[41] Gyrd-Hansen M., Meier P. (2010). IAPs: From caspase inhibitors to modulators of NF-kappaB, inflammation and cancer. Nat. Rev. Cancer.

[42] Adams J.M., Cory S. (1998). The Bcl-2 protein family: Arbiters of cell survival. Science.

[43] Chaudhary A.K., Yadav N., Bhat T.A., O’Malley J., Kumar S., Chandra D. (2016). A potential role of X-linked inhibitor of apoptosis protein in mitochondrial membrane permeabilization and its implication in cancer therapy. Drug Discov. Today.

[44] Srivastava S.K., Xiao D., Lew K.L., Hershberger P., Kokkinakis D.M., Johnson C.S., Trump D.L., Singh S.V. (2003). Allyl isothiocyanate, a constituent of cruciferous vegetables, inhibits growth of PC-3 human prostate cancer xenografts in vivo. Carcinogenesis.

[45] Gianni-Barrera R., Trani M., Reginato S., Banfi A. (2011). To sprout or to split? VEGF, Notch and vascular morphogenesis. Biochem. Soc. Trans..

[46] Scartozzi M., Faloppi L., Svegliati Baroni G., Loretelli C., Piscaglia F., Iavarone M., Toniutto P., Fava G., De Minicis S., Mandolesi A. (2014). VEGF and VEGFR genotyping in the prediction of clinical outcome for HCC patients receiving sorafenib: The ALICE-1 study. Int. J. Cancer.

[47] Sun T., Zhao N., Zhao X.L., Gu Q., Zhang S.W., Che N., Wang X.H., Du J., Liu Y.X., Sun B.C. (2010). Expression and functional significance of Twist1 in hepatocellular carcinoma: Its role in vasculogenic mimicry. Hepatology.

[48] Takeshita N., Hasegawa M., Sasaki K., Seki D., Seiryu M., Miyashita S., Takano I., Oyanagi T., Miyajima Y., Takano-Yamamoto T. (2017). In vivo expression and regulation of genes associated with vascularization during early response of sutures to tensile force. J. Bone Miner. Metab..

[49] Li L.N., Zhou X., Gu Y., Yan J. (2013). Prognostic value of MMP-9 in ovarian cancer: A meta-analysis. Asian Pac. J. Cancer Prev..

[50] Lakka S.S., Gondi C.S., Yanamandra N., Dinh D.H., Olivero W.C., Gujrati M., Rao J.S. (2003). Synergistic down-regulation of urokinase plasminogen activator receptor and matrix metalloproteinase-9 in SNB19 glioblastoma cells efficiently inhibits glioma cell invasion, angiogenesis, and tumor growth. Cancer Res..

[51] Earnshaw W.C., Martins L.M., Kaufmann S.H. (1999). Mammalian caspases: Structure, activation, substrates, and functions during apoptosis. Annu. Rev. Biochem..

[52] Cain K. (2003). Chemical-induced apoptosis: Formation of the Apaf-1 apoptosome. Drug Metab. Rev..

[53] Garrido C., Galluzzi L., Brunet M., Puig P.E., Didelot C., Kroemer G. (2006). Mechanisms of cytochrome c release from mitochondria. Cell Death Differ..

[54] Kumar A., D’Souza S.S., Tickoo S., Salimath B.P., Singh H.B. (2009). Antiangiogenic and proapoptotic activities of allyl isothiocyanate inhibit ascites tumor growth in vivo. Integr. Cancer Ther..

[55] Chou Y.C., Chang M.Y., Wang M.J., Yu F.S., Liu H.C., Harnod T., Hung C.H., Lee H.T., Chung J.G. (2015). PEITC inhibits human brain glioblastoma GBM 8401 cell migration and invasion through the inhibition of uPA, Rho A, and Ras with inhibition of MMP-2, -7 and -9 gene expression. Oncol. Rep..

[56] Chen W.T., Chen Y.K., Lin S.S., Hsu F.T. (2018). Hyperforin Suppresses Tumor Growth and NF-kappaB-mediated Anti-apoptotic and Invasive Potential of Non-small Cell Lung Cancer. Anticancer Res..

[57] Weng M.C., Wang M.H., Tsai J.J., Kuo Y.C., Liu Y.C., Hsu F.T., Wang H.E. (2018). Regorafenib inhibits tumor progression through suppression of ERK/NF-kappaB activation in hepatocellular carcinoma bearing mice. Biosci. Rep..

[58] Huang Y.C., Kuo C.L., Lu K.W., Lin J.J., Yang J.L., Wu R.S., Wu P.P., Chung J.G. (2016). 18alpha-Glycyrrhetinic Acid Induces Apoptosis of HL-60 Human Leukemia Cells through Caspases- and Mitochondria-Dependent Signaling Pathways. Molecules.

[59] Euhus D.M., Hudd C., LaRegina M.C., Johnson F.E. (1986). Tumor measurement in the nude mouse. J. Surg. Oncol..

[60] Ni W.Y., Lu H.F., Hsu S.C., Hsiao Y.P., Liu K.C., Liu J.Y., Ji B.C., Hsueh S.C., Hung F.M., Shang H.S. (2014). Phenethyl isothiocyanate inhibits in vivo growth of subcutaneous xenograft tumors of human malignant melanoma A375.S2 cells. In Vivo.

[61] Liu Y.C., Tsai J.J., Weng Y.S., Hsu F.T. (2020). Regorafenib suppresses epidermal growth factor receptor signaling-modulated progression of colorectal cancer. Biomed. Pharmacother..

[62] Tsai J.J., Hsu F.T., Pan P.J., Chen C.W., Kuo Y.C. (2018). Amentoflavone Enhances the Therapeutic Efficacy of Sorafenib by Inhibiting Anti-apoptotic Potential and Potentiating Apoptosis in Hepatocellular Carcinoma In Vivo. Anticancer Res..

